# Office Lights

**Published:** 1883-03

**Authors:** 


### Office Lights.

Few matters are more important to the furniture of an office than a good light. For this purpose, we can speak with special favor of the "Perfection Student Lamps," devised by Mr. R. G. Hutchinson, 44 Maiden Lane, New York City. They have all the appliances to secure entire safety, are handsome, strong, and the cheapest for the money which we have anywhere seen. Any of our readers who want a really good lamp will do well to send to Mr. Hutchinson for one of his descriptive circulars with particulars.

By a law which has just come into operation in Italy, the sale of patent medicines throughout the kingdom is prohibited unless the precise composition of the medicine is stated. The promulgation of this important decree has been made by the Minister of the Interior, the Customs, and the sanitary authorities. One well-known chemist in Rome has at the present moment nearly $500 worth of patent medicines lying at the Dogana, and likely to have to remain there or to be sent back to England undelivered. For the future travellers will have to smuggle their favorite drugs into Italy.

